# Translation and Validation of the Arrhythmia-Specific Questionnaire in Tachycardia and Arrhythmia (ASTA) to the Brazilian Context: An Instrument Focusing on Arrhythmia Symptoms

**DOI:** 10.1155/2020/1402916

**Published:** 2020-04-10

**Authors:** Priscila M. S. Cannavan, Fernando P. S. Cannavan, Ulla Walfridsson, Maria H. B. M. Lopes

**Affiliations:** ^1^School of Nursing, University of Campinas, Campinas 13083-970, Brazil; ^2^Department of Cardiology, Clinical Hospital, University of Campinas, Campinas 13083-970, Brazil; ^3^Department of Cardiology, University Hospital, Sweden and Department of Medical and Health Sciences, Linköping University, Linköping, 58185, Sweden

## Abstract

**Introduction:**

The wide variety of symptoms in patients with cardiac arrhythmias can affect daily living activities. The evaluation of symptoms with patient-reported outcome measures (PROMs), with validated instruments, can provide information that contributes to clinical decisions and treatment. In Brazil, however, there is no available scale that evaluates symptoms in different types of arrhythmias.

**Purpose:**

This study aimed to translate the Arrhythmia-Specific Questionnaire in Tachycardia and Arrhythmia symptom scale (ASTA-symptom scale) and then validate the questionnaire in terms of Brazilian culture.

**Method:**

The methodological process of cultural adaptation used was based on international literature guidelines consisting of forward translation, synthesis, back translation, review by an expert committee, and pretest. Psychometric analyses were conducted with 140 patients. These included measuring internal consistency (Cronbach's alpha), construct validity with item-total correlations, and convergent construct validity with correlations with the quality of life questionnaire for patients with atrial fibrillation-version 2 (QVFA-v2). Usability and understandability were evaluated through the usability evaluation of instruments.

**Results:**

The translation and adaptation processes were performed by obtaining the Brazilian Portuguese version of the original Swedish instrument. This version presented the internal consistency of items, evaluated through Cronbach's *α* (0.79). Construct validity was demonstrated by item-total correlations for the nine items, all except one reached the level of >0.30 (0.24). Convergent validity showed a high correlation with QVFA-v2 (0.89). As for the evaluation of usability and understanding, after two small suggested changes, no additional alterations were necessary.

**Conclusion:**

The psychometric properties of the Brazilian version of ASTA-symptom scale evaluated in this study were satisfactory, and the scale was proved to be a valid and reliable tool to assess the symptom burden in patients with different forms of tachyarrhythmia. The ASTA-Br-symptom scale questionnaire can be an important addition to PROMs for patients with arrhythmias and could help healthcare professionals in decision-making.

## 1. Introduction

There are several forms of tachyarrhythmia. The classification depends on both characteristics and mechanisms, and they can occur in persons with or without severe heart disease, depending on their characteristics and the mechanism of formation and propagation. Tachyarrhythmia may also occur in individuals with an anatomically normal heart, as well as in those with severe heart disease. Ventricular premature beats and atrial fibrillation (AF) are the most common arrhythmias, the latter occurring in 3% of the general population, with increasing prevalence in elderly patients [1–5].

The most common symptoms in patients with tachyarrhythmia are palpitations, dyspnoea, dizziness, and chest pain. Symptoms such as anxiety and depression, which are less specific, are also mentioned [5–10].

Regardless of the type of arrhythmia, the manifestations presented by the patients can be both physically and mentally related. This can compromise the performance of normal daily life activities and can have a significant negative impact on quality of life (QoL) [5–9, 11, 12].

The assessment of symptoms with disease-specific patient-reported outcome measures (PROMs) can be a valuable aid when making therapeutic decisions [13]. Therefore, in order to properly evaluate the symptoms, a reliable and valid instrument is required. Most of the instruments aimed at PROMs in patients with arrhythmias which are limited to individuals with AF [14–19]. The Arrhythmia-Specific Questionnaire in Tachycardia and Arrhythmia (ASTA) was developed and validated by a Swedish group and was the first disease-specific questionnaire evaluating both symptoms and health-related quality of life (HRQOL) in patients with different arrhythmia diagnoses. It is divided into two specific scales, the ASTA-symptom scale and the ASTA-HRQOL scale, which can be applied together or separately when evaluating the patient with arrhythmia. It also assesses the different forms of tachyarrhythmia, which make it possible to compare patients with supraventricular and ventricular arrhythmias [20, 21]. The internal consistency of the ASTA-symptom scale was evaluated using Cronbach's alpha coefficient, construct validity with item-total correlations and convergent and discriminant validity, and concurrent validity was evaluated. Satisfactory results were obtained for all the items [20]. The ASTA questionnaire has been translated into Spanish and Polish and validated in their respective languages [22, 23].

Due to the variety of symptoms and the effects they may have in patients with tachyarrhythmia, this need to be adequately evaluated, but there is no reliable and valid instrument in Brazil for evaluating symptoms. The reliability and validity of the ASTA-symptom scale have been evaluated in other languages, so this scale can also be used in Brazil. However, due to cultural differences between the countries that validated this instrument and Brazil, a translation, adaptation, and validation process is required. Thus, the purpose of this study was to translate, adapt, and evaluate the psychometric properties of the ASTA-symptom scale to the Brazilian culture and also to evaluate its usability and understandability in patients with different forms of tachyarrhythmia.

## 2. Methods

### 2.1. Study Design and Data Collection

This was a methodological study [24], with the purpose of translating, adapting, and validating the ASTA-symptom scale. Data were collected at the arrhythmology clinic of a public university hospital and at a private clinic specialising in the care of patients with cardiac arrhythmias, both located in the interior of the State of São Paulo.

### 2.2. Ethical Aspects

The use of the ASTA-symptom scale was previously authorised by the author of the questionnaire, and the research was approved by the Research Ethics Committee of the University of Campinas (Unicamp) (CAAE: 78539617.7.0000.5404). All participants that agreed to participate signed the informed consent form.

Patients were invited and advised of their voluntary participation in the research and informed that if they withdrew their consent, this would not have any negative effect on their treatment.

### 2.3. Translation and Adaptation Procedure of the ASTA-Symptom Scale

The ASTA-symptom scale is an instrument developed in Sweden, with nine items evaluating specific symptoms in patients with different types of cardiac arrhythmias. Scores vary from 0 to 27 on a four-point response scale with Likert-type response alternatives ranging from 0 (No) to 3 (Yes, a lot). The scores can be recalculated from 0 to 100, where lower scores characterise fewer symptoms related to arrhythmias [20].

The guidelines for the translation and cultural adaptation of the self-report measures, as recommended by Beaton et al. [25], were used in this study. After the author's formal authorisation, the translation process was performed.

The original translation was carried out by two independent translators, born in Brazil, whose native language was Brazilian Portuguese, who were fluent in Swedish, and who were currently living in Sweden. Translator 1 (T1) was aware of the objectives of the study, while translator 2 (T2) was not. For the synthesis of the translations (T1 and T2), the versions were shown to the researcher and a mediator, specialist in arrhythmia. The discrepancies between T1 and T2 were analysed and synthesised into a single version of the questionnaire (T12).

The translation of the ASTA-symptom scale back into the original language was carried out by two independent Swedish translators with knowledge of both Brazilian Portuguese language and culture. At this stage, the translators were not aware of the purpose of the study. The synthesis of the version in Portuguese (T12) was made available for the backward translation, which was then translated into the original Swedish language. This translation was the origin of versions BT1 and BT2.

All the previously produced instruments (T1, T2, T12, BT1, and BT2) were reviewed and analysed by a committee of experts that evaluated the equivalences: 1. semantics—maintenance of the meaning of each item after translation into Brazilian Portuguese; 2. idiomatic—adequate translation of colloquial expressions from Swedish to Portuguese; 3. culture—consistency between the terms used in the original version and the corresponding one in Brazilian Portuguese; and 4. conceptual—equivalence between the different conceptual meanings of different cultures, with maintenance of the coherence between the item and the domain to be evaluated. The judges were asked to evaluate whether the situations evoked or portrayed in the items actually assessed the symptom burden.

The committee was composed of eight members: four translators fluent in both languages, a Brazilian nurse with clinical experience in the area of cardiology in Sweden, a methodologist (a nurse practitioner with knowledge of the theoretical framework), a linguist with knowledge of the Portuguese language, and a physician specialist in cardiac arrhythmias from the Brazilian Society of Cardiac Arrhythmias.

The individual assessment of semantic, idiomatic, cultural, and conceptual equivalence by each member of the committee was performed in two steps: (1) judges scored each item on a Likert scale, with a score of 1 to 4, where 1 indicated no equivalence; 2, it was impossible to evaluate the equivalence without the item being revised; 3 equivalent, but needed minor changes; and 4 completely equivalent. In the same worksheet, the judges still had to evaluate the comprehensiveness and relevance of the questionnaire. All the judges were previously advised on how they should proceed in the evaluation. In this first stage, the content validity index, which measures the agreement between the evaluators, was considered acceptable when equal to or greater than 80% for each item of the instrument [26]. (2) After the first analysis, if necessary, the translated questionnaire was modified, according to suggestions submitted by the judges, ideally aiming to achieve 100% agreement by consensus [26]. At the end, the preliminary version of the questionnaire to be used in the pretest was obtained.

For the pretest, 30–40 participants who met the inclusion criteria and also agreed to participate in the study were interviewed. All participants were informed about the purpose of the study, and the usability and understandability of the translated instrument were assessed using the usability evaluation of instruments [27].

### 2.4. Validity and Reliability of the Brazilian Portuguese Version of the ASTA-Symptom Scale

#### 2.4.1. Patient Selection

A nonprobabilistic convenience sample of patients with a tachyarrhythmia diagnosis was selected. The sample size would be equivalent to a minimum of 10 respondents for each item of the instrument (ASTA-symptom scale) following literature recommendations [28].

The inclusion criteria were people aged 18 or more, diagnosed with tachyarrhythmia for more than three months, without an implantable electronic cardiac device, score equal to or greater than 5 on the Short Portable Mental Status Questionnaire (SPMSQ) [29], and who agreed to participate in the study after being informed of its purpose.

#### 2.4.2. Study Protocol

Before the medical consultation, the researcher initiated contact and explained the research project. Interviews and data collection took place in a private room. The data were collected by the researcher through a tablet. Each question and related options were read to the patients so that they could indicate their answers. The following instruments were used: the sample characterisation record (developed for this study), the Brazilian Portuguese version of the ASTA-symptom scale, and the quality of life questionnaire for patients with atrial fibrillation-version 2 (QVFA-v2) [18].

The Sample characterisation record had the purpose of delineating the profile of the sample studied, containing both sociodemographic and clinical information. The QVFA was developed and validated for evaluation of clinical manifestations and treatment of patients with AF [18]. In 2016, Moreira et al. [19] developed version 2 (QVFA-v2) excluding domains related to therapeutics and included the domains: fatigue, perception of disease, and well-being and retained the domains: palpitations, dyspnoea, chest pain, and dizziness. The QVFA-v2 consists of seven domains and 30 questions. All domains have the same score, 20 points, giving a maximum score of 140, where higher scores represent a more negatively affected QoL by symptoms [19].

To perform the collection and data management, the REDCap (Research Electronic Data Capture) [30] platform, a secure Web-based data capture application, was used and hosted on the server of the Faculty of Medical Sciences of Unicamp.

#### 2.4.3. Data Analyses

Means, standard deviations, and frequencies were used to describe patient characteristics and symptoms. The Brazilian Portuguese version of the ASTA-symptom scale (ASTA-Br-symptom scale) was psychometrically evaluated regarding data quality, construct validity, and internal consistency reliability.

The convergent validity of the ASTA-Br-symptom scale with the QVFA-v2 questionnaire was evaluated using Spearman's correlation coefficient [31]. Cohen [32] suggests the following classification of the correlation coefficient: 0.1 to 0.29 (weak), 0.30 to 0.49 (moderate), and greater than or equal to 0.50 (strong).

The hypothesis tested was that there was a correlation between ASTA-Br-symptom and the following domains of the QVFA-v2: palpitations, dyspnoea, chest pain, dizziness, and fatigue.

The internal consistency analysis of the instruments was evaluated using Cronbach's alpha coefficient, where *α* coefficient ≥0.70 was considered sufficient. Construct validity was supported by item-total correlations above the acceptable level ≥0.30. [33, 34]. The analyses were performed using software SAS 9.4 and SPSS 22.

## 3. Results

### 3.1. Cultural Adaptation

There were no recommendations from the expert committee regarding modifications for clarification/explanation. In order to facilitate usability, the layouts for some questions were redesigned.

For the pretest, the ASTA-Br-symptom scale was applied to a sample of 32 participants with tachyarrhythmia. Along with the ASTA-Br-symptom scale, the Usability Assessment Questionnaire [27] was answered by the participants in order to evaluate the usability of the ASTA-Br-symptom scale, that is, the understanding of instructions and requests and how to answer them. The majority (93.8%) of the respondents reported that the items were easy to understand.

### 3.2. Patient Demographics

Data were collected between May and October, 2018. For psychometric evaluation, 140 patients with a diagnosis of cardiac arrhythmias were included, with a mean age of 57.2 years (SD 13.1), 55% (*n* = 77) female, 62.14% (*n* = 87) studied until elementary school, 79.28% (*n* = 111) had remuneration of 1 to 2 minimum wages, and only 32.14% (*n* = 45) were employed. Of these, 135 (96.4%) participants came from the outpatient clinic of the public service.

Regarding clinical characteristics, 129 (92.1%) participants had a diagnosis of supraventricular tachyarrhythmia and 11 (7.9%) had ventricular arrhythmias, with a mean diagnostic time of 106.5 months (SD 112.1), a minimum of three months and a maximum of 42 years, and 118 (84.3%) were not aware of the type of arrhythmia. Participants with associated cardiovascular disease (62.14%) had a mean left ventricular ejection fraction (LVEF) of 60% (SD 10.7) obtained from the echocardiographic data in the medical record.  Table 1 presents the clinical characteristics of the participants.

As to the onset of symptoms, 62 (44.3%) patients reported that the symptoms started during physical effort and 25 (17.9%) either in situations of stress or emotion; 78 (55.7%) had come close to fainting in connection with arrhythmia, and 35 (25%) had fainted in connection with arrhythmia; 107 (76.4) reported that the symptoms lasted on average less than one hour; and 43 (30.7%) reported having symptoms every day.

Regarding the specificity of the palpitations, the patients were asked and could choose more than one alternative, and 122 (87.1%) reported a feeling that the heart was beating fast, 101 (72.1%) felt the heart beating harder than usual, 57 (40.7%) felt the heart beating irregularly, and 33 (23.6%) reported a feeling that the heart missed one or more beats.

### 3.3. Internal Consistency Reliability

As in the original version [20], the internal consistency of the Brazilian version presented results with sufficient homogeneity, Cronbach's alpha = 0.79. All nine items demonstrated satisfactory internal consistency, with Cronbach's alpha values ranging from 0.75 to 0.80 if the item was deleted (Table 2).

#### 3.3.1. Construct Validity

The item-total correlations for the nine items in the ASTA-Br-symptom scale reached the level of >0.30, ranging from 0.41 (cold sweat) to 0.61 (weakness/fatigue), all except for worry/anxiety, 0.24 (Table 2).

#### 3.3.2. Convergent Construct Validity

The correlation between “ASTA-Br-symptom” scores and “QVFA-v2” questionnaires evaluated by the Spearman correlation coefficient was 0.89 (*p*  < 0.0001), showing a strong positive and significant correlation.

## 4. Discussion

The translated and adapted ASTA-Br-symptom scale demonstrated generally good reliability and validity properties. The study was conducted using a rigorous methodology, with a sample that included participants of both sexes, with atrial and ventricular arrhythmias and with a sample size adequate for the psychometric tests used.

The ASTA-symptom scale proposes to evaluate the number of symptoms in patients with different forms of tachyarrhythmia. A thorough review of the literature showed that the ASTA questionnaire is the first one specifically developed to evaluate symptoms in patients with different arrhythmias, making comparisons easy between the different diagnoses. It was also found that this is the only study that reports the use of validated scales in Brazil to assess symptoms in this population.

The adaptation of an existing scale is considered more appropriate than developing new ones for the same construct, and there are significant advantages since the researcher can compare data obtained in different scenarios [35].

The use of disease-specific questionnaires is increasingly frequent since it subsidises health decision-making. However, for their wider use, it is necessary to adapt them for different cultures. For this to be possible, the process of cultural adaptation must take place with methodological rigour to maintain the validity and reliability of the questionnaires [36].

Reliability refers to the degree to which an instrument produces consistent results from its scores. When one item is eliminated and Cronbach's alpha coefficient increases, this item is not highly correlated with the others and could be eliminated from the instrument, but if the coefficient decreases, it means that the item is highly correlated with the others [37]. The internal consistency value by Cronbach's alpha was equal to the value obtained in the original instrument. In this study, the item worry/anxiety was borderline and increased Cronbach's alpha coefficient if deleted [20].

Construct validity was supported by the item-total correlations above the acceptable level (≥0.30) for all except one of the items, worry/anxiety. However, this did not lead to a withdrawal from the ASTA-Br-symptom scale.

Regarding construct validity, there was a strong correlation between the ASTA-Br-symptom scale and QFVA-v2 questionnaire, supporting convergent validity.

In the sample, there were no patients with a significant reduction in LVEF, showing that the symptoms eventually referred to as breathlessness, weakness/fatigue, and tiredness may be directly related to the presence of arrhythmia. It should be noted that when asked about these symptoms, the participant should relate them to the arrhythmia, which could minimise the influence of other conditions that cause these symptoms on the participant's response.

The majority of the respondents found the items easy to understand, and the evaluation of usability and understandability only led to a few changes, suggested by the patients.

### 4.1. Methodological Considerations/Limitations

The study had some limitations. One of them was that there were few patients diagnosed with atrial tachycardia or with ventricular arrhythmias. Half of the patients were diagnosed with the most common arrhythmia of all, AF. The majority of respondents had low schooling and low income and were included from two areas with different sociocultural characteristics. In addition, due to the country's geographic size and also because of the economic, cultural, and social specificities of each region, perhaps this sample is not representative of the total Brazilian population living with tachyarrhythmia. Therefore, more studies are recommended that include the different regions of the country.

## 5. Conclusions

The psychometric properties of the Brazilian version of the ASTA-symptom scale evaluated in this study were satisfactory, and the scale was proved to be a valid and reliable tool to assess the symptom burden in patients with different forms of tachyarrhythmia. The ASTA-Br-symptom scale questionnaire can be an important addition to PROMs in patients with arrhythmias and can help healthcare professionals in decision-making.

## Figures and Tables

**Table 1:** Clinical characteristics of the participants (*n* = 140).

Variables	*N*	%	LVEF

Type of cardiac arrhythmia			
Atrial fibrillation	71	50.71	
Atrial flutter	19	13.57	
Atrial tachycardia	2	1.43	
Paroxysmal supraventricular tachycardia	7	5.00	
Wolff–Parkinson–White	14	10.00	
AV nodal re-entry	16	11.43	
Ventricular tachycardia	1	0.71	
Ventricular extrasystoles	6	4.29	
Nonsustained ventricular tachycardia	4	2.86	

Treatment^*∗*^			
Medication	132	94.29	
Catheter ablation	17	12.14	
DC electrical cardioversion	5	3.57	
Hospitalisation	78	55.71	
Associated cardiovascular disease	87	62.14	
Antiarrhythmic medication	122	87.14	
Class I	10	7.14	
Class I	91	65.00	
Class III	18	12.86	
Class IV	3	2.14	
Oral anticoagulant	77	55.00	

NYHA functional class			
I	96	68.57	62 (SD 9.91)
II	43	30.71	55 (SD 13.15)
III	1	0.71	60 (SD 0)

Cardiovascular diseases^*∗*^			
Hypertension	69	49.29	
Cardiac valve replacement	16	11.43	
Dyslipidaemia	16	11.43	
Chagas disease	10	7.14	
Dilated cardiomyopathy	2	1.43	
Diabetes mellitus	32	22.86	

Other clinical characteristics and comorbidity^*∗*^			
Hypothyroidism	18	12.86	
Systemic lupus erythematosus	3	2.14	

LVEF = left ventricular ejection fraction. ^*∗*^Participants could choose more than one answer option.

**Table 2:** Data quality and item-total correlations for the ASTA-Br-symptom scale.

Items	Symptom scale			Response alternatives			
Symptoms	Item-total correlation	Cronbach if item was deleted	Mean (SD)	No, *n* (%)	Yes, to a certain extent *n* (%)	Yes, quite a lot *n* (%)	Yes, a lot *n* (%)
Breathlessness during activity	0.42	0.78	1.54 (1.14)	39 (27.86)	21 (15.00)	46 (32.86)	34 (24.29)
Breathlessness even at rest	0.51	0.77	0.99 (1.10)	64 (45.71)	33 (24.57)	23 (16.43)	20 (14.29)
Dizziness	0.45	0.78	0.96 (1.04)	63 (45.00)	34 (24.29)	28 (20.00)	15 (10.71)
Cold sweat	0.41	0.78	0.91 (1.17)	76 (54.29)	25 (17.86)	14 (10.00)	25 (17.86)
Weakness/fatigue	0.61	0.75	1.12 (1.18)	62 (44.29)	27 (19.29)	23 (16.43)	28 (20.00)
Tiredness	0.58	0.76	1.44 (1.21)	46 (32.86)	25 (17.86)	30 (21.43)	39 (27.86)
Chest pain	0.55	0.76	0.93 (1.20)	78 (55.71)	21 (15.00)	14 (10.00)	27 (19.29)
Pressure/discomfort in the chest	0.56	0.76	1.07 (1.15)	62 (44.29)	31 (22.14)	22 (15.71)	25 (17.86)
Worry/anxiety	0.24	0.80	1.48 (1.35)	58 (41.43)	9 (6.43)	21 (15.00)	52 (37.14)
—Total cronbach		**0.79**	**10.45 (6.44)**				

ASTA-Br-symptom scale: Arrhythmia-Specific Questionnaire in the Tachycardia and Arrhythmia ASTA-Brazilian-Symptom scale.

## Data Availability

The data used to support the findings of this study are available in the Research Electronic Data Capture-REDCap platform hosted on the server of the Faculty of Medical Sciences of Unicamp.
